# Postmortem Diagnosis of Neonatal Hemochromatosis Following Fulminant Multiorgan Failure

**DOI:** 10.7759/cureus.117677

**Published:** 2026-10-09

**Authors:** Takehiro Tanabe, Kenji Sonota, Tanomo Ono, Daiki Abukawa, Taku Koizumi

**Affiliations:** 1 Critical Care, Miyagi Children's Hospital, Miyagi, JPN; 2 Gastroenterology and Hepatology, Miyagi Children's Hospital, Miyagi, JPN

**Keywords:** extrahepatic siderosis, gestational alloimmune liver disease, magnetic resonance imaging, neonatal acute liver failure, neonatal hemochromatosis

## Abstract

Neonatal hemochromatosis (NH) is a rare cause of neonatal acute liver failure. Hepatic injury disrupts iron homeostasis, including hepatic hepcidin regulation, resulting in systemic iron overload and iron deposition in extrahepatic tissues. However, diagnosing it remains challenging because clinical manifestations are nonspecific and histological confirmation may be difficult to obtain. Herein, we report a fatal case of NH in a term neonate who developed fulminant multiorgan failure shortly after birth, followed by a prolonged critical course. The patient developed severe metabolic acidosis, coagulopathy, anuric renal failure requiring continuous renal replacement therapy, and persistent cardiovascular instability despite intensive treatment. Magnetic resonance imaging showed characteristic extrahepatic siderosis with sparing of the spleen. Although salivary gland biopsy findings were negative, the autopsy confirmed widespread iron deposition consistent with NH. Extensive investigations excluded metabolic, mitochondrial, and lysosomal disorders, including Niemann-Pick disease type C. This case highlights the diagnostic difficulty of NH and the importance of early recognition and comprehensive evaluation in neonates with acute liver failure.

## Introduction

Neonatal hemochromatosis (NH) is a rare but life-threatening disorder characterized by liver failure and extrahepatic iron deposition [1,2]. It is now widely recognized as a manifestation of gestational alloimmune liver disease, in which maternal immunoglobulin G antibodies target fetal hepatocytes, resulting in severe fetal liver damage and dysregulated iron metabolism [3,4]. Early diagnosis is clinically important because timely recognition may allow consideration of liver transplantation, whereas confirmation of the diagnosis has important implications for counseling on recurrence risk and antenatal intravenous immunoglobulin (IVIG) therapy in subsequent pregnancies [4,5]. However, diagnosis remains challenging because clinical manifestations are nonspecific and histological confirmation may be difficult to obtain [6,7].

Herein, we report a fatal case of NH in a term neonate with fulminant multiorgan failure, in whom the diagnosis was established after postmortem examination based on characteristic magnetic resonance imaging findings, autopsy confirmation, and comprehensive exclusion of metabolic, mitochondrial, and lysosomal disorders, including Niemann-Pick disease type C.

## Case presentation

A baby girl was born at 40 weeks of gestation via vaginal delivery (birth weight 3,494 g; Apgar scores 8 and 9 at 1 and 5 min, respectively) to a healthy 32-year-old woman (gravida 2, para 1) with no relevant medical history. The mother had previously borne a healthy boy after an uneventful pregnancy. Although the current patient was fed twice after birth, she developed hematemesis and respiratory distress at 9 hours of life. At a regional neonatal intensive care unit, laboratory evaluation at 12 hours of life revealed profound hypoglycemia, lactic acidosis, and severe metabolic acidosis. She was transferred to our center and admitted to the pediatric intensive care unit at 15 hours of life.

On admission, hepatomegaly was absent. Continuous renal replacement therapy was initiated within 3 hours of admission because of refractory severe metabolic acidosis. During the subsequent clinical course, the patient became anuric and remained dialysis-dependent. Severe coagulopathy precluded the initial measurement of coagulation factor levels and persisted despite repeated fresh-frozen plasma transfusions. Continuous vasopressor support was required, and hemodynamic stability could not be maintained without pharmacological support. Laboratory tests revealed markedly high lactate and pyruvate levels, hyperferritinemia, and iron overload (Table 1).

**Table 1:** Laboratory data at admission The blood gas sample taken at 12 hours of life is venous blood. pCO_2_, partial pressure of carbon dioxide; pO_2_, partial pressure of oxygen; WBC, white blood cell; PT-NNR, prothrombin time-international normalized ratio; APTT, activated partial thromboplastin time; CK, creatine kinase; TBIL, total bilirubin; LDH, lactate dehydrogenase; AFP, alpha-fetoprotein

Parameters	Patient values at NICU (12 h after birth)	Patient values at PICU (15 h after birth)	Reference range
pH	7.107	6.993	7.35–7.45
pCO_2_ (mmHg)	15	24.9	35–45
pO_2_ (mmHg)	89.1	211	80–100
Bicarbonate (mmol/L)	4.7	5.8	22–28
Base excess (mmol/L)	-22.2	-24.1	-2 to +2
Anion gap (mmol/L)	28.3	26.9	8–16
Glucose (mg/dL)	4	133	70–99
WBC (×10^3^/μL)	-	48.3	4.0–10.0
PT-INR	-	>5.0	0.9–1.1
APTT (s)	-	>180	25–35
CK (U/L)	-	1,647	40–200
Lactate (mg/dL)	-	211.1	4.5–18
Pyruvic acid (mg/dL)	-	3.6	0.3–0.9
TBIL (mg/dL)	-	5.19	0.2–1.2
LDH (U/L)	-	938	120–240
Creatinine (mg/dL)	-	1.21	0.5-0.9
Iron (μg/dL)	-	327	60–170
Ferritin (ng/ml)	-	7,350.2	20–200
AFP (ng/mL)	-	3,900.1	10,000–1100,000

Despite intensive care, including exchange transfusion and IVIG initiated at approximately 3 weeks of life, liver and renal dysfunction persisted. Liver transplantation was considered from the second week of life and evaluated over the following month; however, she was ultimately deemed unsuitable for transplantation because of persistent severe multiorgan dysfunction and poor overall clinical condition. She died on day 61 of life.

Magnetic resonance imaging (MRI) (Figure 1) on day 41 depicted T2* signal loss in the pancreas, thyroid, pituitary, and pineal glands, with sparing of the spleen.

**Figure 1:**
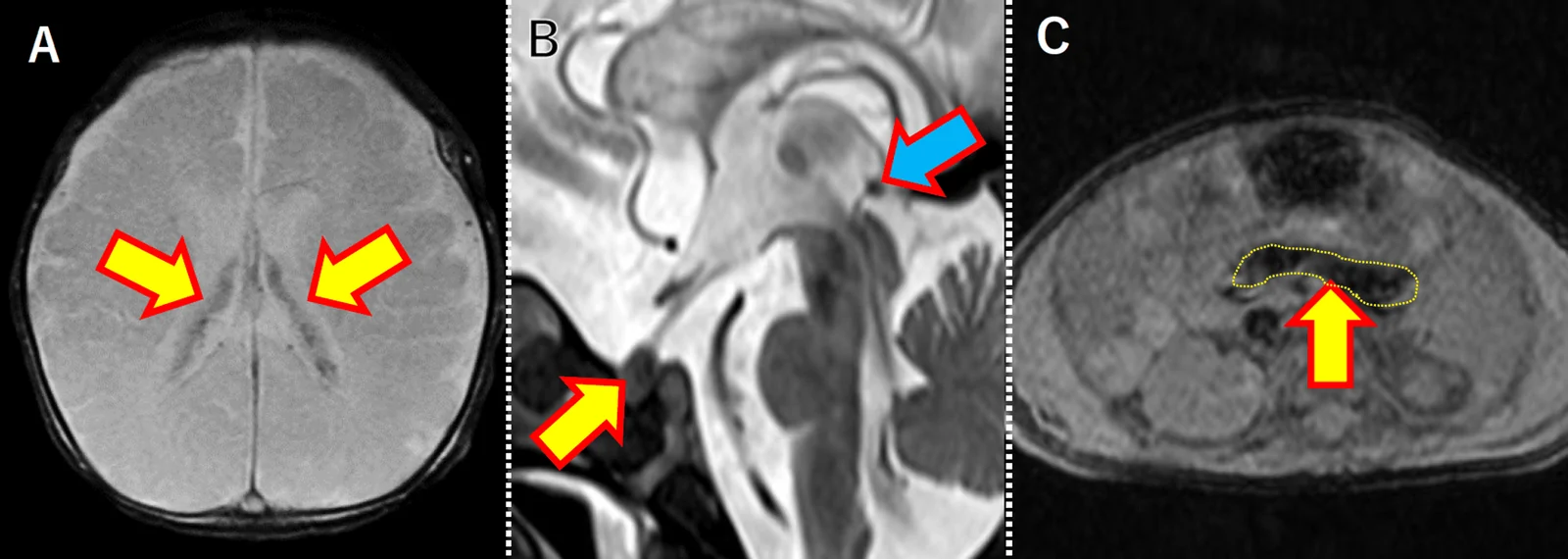
Magnetic resonance imaging (A) T2*-weighted image of the brain, revealing signal loss in the choroid plexus (arrow), indicative of iron deposition. (B) T2-weighted image of the brain, indicating signal loss in the anterior pituitary gland (yellow arrow) and pineal gland (blue arrow), consistent with iron deposition. (C) T2-weighted image of the abdomen, revealing substantial signal loss in the pancreas (arrow), suggestive of iron overload.

Salivary gland biopsy showed no iron deposition. Autopsy (Figure 2) revealed widespread iron deposition in multiple organs, with sparing of the spleen, kidneys, lungs, and esophagus, and giant-cell transformation of the liver.

**Figure 2:**
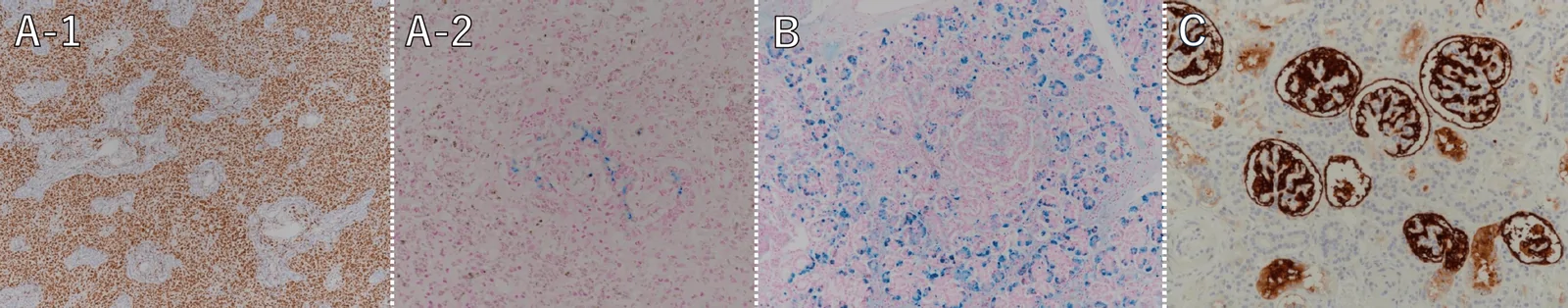
Histopathological findings (A) Liver specimen, revealing several CD68-positive macrophages (A-1), suggesting widespread hepatocellular necrosis. Iron staining (Prussian blue) highlighted iron-positive macrophages within the bile ducts, presumably due to hepatocyte loss (A-2). (B) The pancreas reveals widespread iron deposition on iron staining. (C) Kidney specimen: CD10 immunostaining revealed only a few positive tubular cells, suggesting reduced proximal tubular structures. CD, cluster of differentiation

Given the persistent liver failure of unknown etiology, extensive investigations for alternative diagnoses were performed. Urinary organic acid analysis and tandem mass spectrometry did not suggest a specific inborn error of metabolism. Whole-genome sequencing identified no pathogenic variant that could explain the clinical course. Mitochondrial disease was investigated by measuring respiratory chain enzyme activities in hepatic tissue and other tissues. Mild reductions in complex II and III activities were observed in hepatic tissue; however, complex IV activity and citrate synthase levels were preserved, and respiratory chain enzyme activities in fibroblasts, skeletal muscle, heart, and kidney were within normal ranges. These findings did not support a primary mitochondrial disorder. Niemann-Pick disease type C was evaluated using disease-specific biomarkers, including urinary bile acids, plasma oxysterols, Lyso-SM509, and lysosphingomyelin, all of which were within normal limits, and sequencing of NPC1 and NPC2 revealed no pathogenic variants. Based on the clinical, radiological, pathological, biochemical, and genetic findings, NH was diagnosed after postmortem examination.

## Discussion

NH is a rare but often fatal condition characterized by neonatal liver failure and extrahepatic iron deposition, typically with sparing of the reticuloendothelial system [1,2]. The present case involved several important clinical and diagnostic features. The patient developed fulminant liver failure and multiorgan dysfunction within hours after birth, without hepatomegaly, and remained critically ill despite maximal supportive care. The relatively low AFP level despite severe liver injury and iron overload was also suggestive of NH. MRI demonstrated a characteristic extrahepatic siderosis pattern, with T2 hypointensity in the pancreas and endocrine organs, with sparing of the spleen, consistent with previous reports [8]. Although the minor salivary gland biopsy was negative, its limited sensitivity does not exclude the diagnosis [9]. The patient remained dependent on vasopressors, dialysis, and transfusion support, with recurrent sepsis, and was thus ineligible for liver transplantation, underscoring the importance of early recognition before irreversible multiorgan failure develops. The diagnosis was ultimately confirmed at autopsy, which revealed a distribution of iron deposition that closely corresponded with the ante-mortem MRI findings. Pathological examination revealed iron deposition in organs such as the liver, pancreas, thyroid, parathyroid, and heart, whereas no iron deposition was observed in the lungs, kidneys, spleen, or esophagus. The kidneys exhibited hypoplasia of the proximal tubules, suggesting that fetal liver injury impaired hepatic angiotensinogen production. Hepatic angiotensinogen is important for fetal proximal tubular development, and reduced hepatic angiotensinogen expression has been associated with proximal tubular dysgenesis in NH [10].

A notable strength of this case is the comprehensive exclusion of alternative diagnoses, particularly mitochondrial disorders and Niemann-Pick disease type C, both of which can present with similar features [11,12]. Both mitochondrial disease and Niemann-Pick disease type C require enzyme activity assays or genetic testing, and establishing a definitive diagnosis of NH takes time. In the present case, these conditions were evaluated using extensive functional, biochemical, and genetic testing, providing additional support for the diagnosis of NH. Extensive functional, biochemical, and genetic testing provided a high level of diagnostic certainty, which was essential for appropriate family counseling regarding recurrence risk and guidance on preventive strategies [3]. From a therapeutic perspective, this case highlights the challenges of managing severe NH. Although liver transplantation was considered from the second week of life and evaluated over the following month, persistent severe multiorgan dysfunction and poor overall clinical condition ultimately rendered the patient unsuitable for transplantation. NH-directed therapies, including exchange transfusion and IVIG, were initiated at approximately three weeks of life, when NH became a stronger diagnostic consideration; deferoxamine mesylate was also administered. Despite these interventions, multiorgan dysfunction persisted. The relatively delayed recognition of NH may have limited the opportunity for earlier disease-specific treatment and transplantation before irreversible multiorgan failure developed. Importantly, a previously unaffected pregnancy does not exclude gestational alloimmune liver disease, and the diagnosis remains relevant for counseling regarding recurrence risk and preventive antenatal IVIG therapy in subsequent pregnancies [5]. In this case, establishing a definitive diagnosis during the patient's lifetime was particularly challenging because the characteristic pathological findings were confirmed only at autopsy, and several differential diagnostic investigations required time to complete.

An additional educational message from this case is that NH should be considered in the differential diagnosis of neonatal acute liver failure, even when classic findings are absent [6,7]. The patient lacked hepatomegaly and had a negative minor salivary gland biopsy, findings that may have contributed to reduced clinical suspicion for NH. However, markedly elevated ferritin levels, together with the characteristic MRI pattern of extrahepatic siderosis, prompted further investigation. This case, therefore, emphasizes that MRI may provide important diagnostic clues when a tissue biopsy result is non-diagnostic and that a negative salivary gland biopsy should not exclude NH. Clinicians should maintain a high index of suspicion for neonates presenting with acute liver failure, severe coagulopathy, hypoglycemia, and hyperferritinemia, as early recognition may preserve opportunities for disease-specific management and liver transplantation.

A limitation of this case is that C5b-9 immunostaining, which may provide more specific pathological evidence of gestational alloimmune liver disease, was not performed. Therefore, the diagnosis of NH was based on the integration of the characteristic clinical and radiological findings, widespread extrahepatic iron deposition at autopsy, and comprehensive exclusion of alternative diagnoses.

## Conclusions

NH should remain in the differential diagnosis of neonatal acute liver failure, even in the absence of typical features such as hepatomegaly or positive minor salivary gland biopsy findings. Acute liver failure accompanied by severe coagulopathy, hypoglycemia, hyperferritinemia, and characteristic MRI findings should raise suspicion for NH, as early recognition may facilitate timely disease-specific management and evaluation for liver transplantation. Because the diagnosis of NH is often challenging, multidisciplinary integration of clinical, radiological, pathological, and genetic findings is essential to establish diagnostic certainty. Accurate diagnosis is also crucial for appropriate family counseling regarding recurrence risk and consideration of antenatal IVIG therapy in subsequent pregnancies.

## References

[1] Feldman AG, Whitington PF (2013). Neonatal hemochromatosis. J Clin Exp Hepatol.

[2] Shanmugam NP, Bansal S, Greenough A, Verma A, Dhawan A (2011). Neonatal liver failure: aetiologies and management--state of the art. Eur J Pediatr.

[3] Whitington PF (2012). Gestational alloimmune liver disease and neonatal hemochromatosis. Semin Liver Dis.

[4] Whitington PF (2007). Neonatal hemochromatosis: a congenital alloimmune hepatitis. Semin Liver Dis.

[5] Whitington PF, Kelly S, Taylor SA, Nóbrega S, Schreiber RA, Sokal EM, Hibbard JU (2018). Antenatal treatment with intravenous immunoglobulin to prevent gestational alloimmune liver disease: comparative effectiveness of 14-week versus 18-week initiation. Fetal Diagn Ther.

[6] Larson-Nath C, Vitola BE (2020). Neonatal acute liver failure. Clin Perinatol.

[7] Taylor SA, Whitington PF (2016). Neonatal acute liver failure. Liver Transpl.

[8] Chavhan GB, Kamath BM, Siddiqui I, Tomlinson C (2022). Magnetic resonance imaging of neonatal hemochromatosis. Pediatr Radiol.

[9] Smith SR, Shneider BL, Magid M, Martin G, Rothschild M (2004). Minor salivary gland biopsy in neonatal hemochromatosis. Arch Otolaryngol Head Neck Surg.

[10] Bonilla SF, Melin-Aldana H, Whitington PF (2010). Relationship of proximal renal tubular dysgenesis and fetal liver injury in neonatal hemochromatosis. Pediatr Res.

[11] Kumagai T, Terashima H, Uchida H (2019). A case of Niemann-Pick disease type C with neonatal liver failure initially diagnosed as neonatal hemochromatosis. Brain Dev.

[12] Park SW, Park JH, Moon HJ, Shin M, Moon JS, Ko JS (2021). Niemann-Pick disease type C diagnosed using neonatal cholestasis gene panel. Korean J Gastroenterol.

